# Sclerostin Concentration and Bone Biomarker Trends in Patients with Spinal Cord Injury: A Prospective Study

**DOI:** 10.3390/healthcare10060983

**Published:** 2022-05-25

**Authors:** Jong Ho Lee, Jang Hyuk Cho, Dong Gyu Lee

**Affiliations:** 1 Department of Laboratory Medicine, College of Medicine, Yeungnam University, Daegu 42415, Korea; leejongho@ynu.ac.kr; 2 Department of Rehabilitation Medicine, Keimyung University School of Medicine, Keimyung University Dongsan Medical Center, Daegu 42601, Korea; analogcho@hanmail.net; 3 Department of Physical Medicine and Rehabilitation, College of Medicine, Yeungnam University, Daegu 42415, Korea

**Keywords:** CTX, P1NP, spinal cord injury, osteoporosis, sclerostin

## Abstract

Patients with spinal cord injury (SCI) experience a high osteoporosis incidence, which increases fracture risk. Recently, a sclerostin antibody was introduced as a target biomarker to treat osteoporosis. We aimed to determine the serum concentration of sclerostin and factors affecting its concentration over time. This was a prospective cross-sectional study. The inclusion criteria were (1) SCI patients with a grade 3 modified functional ambulatory category score (FAC—patients requiring firm continuous support) and (2) patients whose injury occurred >1 month ago. The exclusion criterion was a history of osteoporosis medication administration within 6 months. The collected data included bone biomarkers (carboxy-terminal collagen crosslinks (CTX), procollagen type 1 intact N-terminal propeptide, and sclerostin), clinical data (FAC, lower extremity motor score), body mass index, SCI duration, and hip bone mineral density (BMD). This study recruited 62 patients with SCI. Sclerostin levels significantly correlated with age, CTX level, and hip BMD. SCI duration was negatively correlated with sclerostin levels. Lower extremity motor scores were not significantly correlated with sclerostin levels. The acute SCI state showed a higher sclerostin level than the chronic SCI state. Sclerostin showed a significant relationship with CTX. In conclusion, age and BMD affect sclerostin concentration in patients with SCI.

## 1. Introduction

Qualified acute care for patients with spinal cord injury (SCI) increases their survival rate following SCI. Rehabilitation therapy for SCI focuses on the prevention and treatment of secondary complications. Osteoporosis is a critical medical problem in patients with SCI [1]. Fifty percent of patients with chronic SCI are at risk of osteoporotic fracture at a long-term follow-up [2]. Femur fractures are associated with a high risk of mortality and decreased quality of life [3]. Osteoporosis in patients with SCI frequently yields distal femur or tibial fractures, which add to the medical and social burden of the patient [4]. Metabolic and endocrine changes in acute SCI, as well as subsequent neurological deficits, influence changes in bone metabolism. In particular, non-ambulatory patients with SCI have a higher incidence of osteoporosis than ambulatory patients [5]. Therefore, unloading is regarded as a critical factor for secondary osteoporosis in patients with SCI. Moreover, since the muscle and bone are linked to a highly disabling condition, changes in bone metabolism in patients with SCI with muscle weakness require more attention [6]. Medication and physical therapy, such as standing using equipment, have been used to treat and prevent osteoporosis [7,8]. However, the prevention and treatment of osteoporosis in patients with SCI remains a challenge.

After spinal cord injury, within several months, bony absorptive metabolism is markedly increased [9]. Therefore, antiabsorptive medications, such as bisphosphonates, have been used to prevent osteoporosis in patients with SCI [10]. However, there is insufficient evidence regarding the effectiveness of bisphosphonates in maintaining bone mineral density (BMD) after acute SCI. Recently, antisclerostin antibodies have been introduced as an effective treatment for osteoporosis [11]. Sclerostin, which is secreted by osteocytes, is a biomarker that modulates bone metabolism [12]. Increased sclerostin levels decrease osteoblast activation, resulting in decreased bone density. In addition, osteocytes are terminally differentiated from osteoblasts and are embedded in the mineralized matrix [13]. Sclerostin is secreted in response to gravity [14]. Accordingly, osteocytes are responsible for converting mechanical strain into biological signals. Unloading following SCI induces osteocytes to secrete sclerostin. Patients with SCI show a higher serum concentration of sclerostin than controls [15]. Unloading is a critical clinical symptom of SCI. Studies on sclerostin will aid in the treatment and prevention of secondary osteoporosis in patients with SCI.

Bone biomarkers measure enzymes secreted by osteoclasts and osteoblasts during bone resorption and bone formation. Therefore, bone biomarkers are indicators that reflect the rate of bone turnover and can be used to evaluate bone quality. Carboxy-terminal collagen crosslinks (CTX) are produced by the degradation of cross-linked peptides in collagen fibers during bone absorption. Procollagen type 1 intact N-terminal propeptide (P1NP) is a peptide from which a propeptide extension of procollagen, created by osteoblasts, is removed. Therefore, CTX and P1NP have been selected as surrogate markers for bone absorption and formation, respectively [16].

Few studies have examined the sclerostin levels in patients with SCI. In this study, we aimed to determine the sclerostin trends following SCI. We also evaluated the relationship between sclerostin and bone biomarkers, hip BMD, and various clinical factors (SCI duration, age, and body mass index [BMI]).

## 2. Materials and Methods

### 2.1. Patients

We prospectively identified patients with SCI between January 2020 and October 2021. This study was approved by the institutional review board (YUMC 2019-11-054) and conducted in accordance with the Declaration of Helsinki guidelines. Written informed consent was obtained from all recruited patients.

This was a prospectively collected cross-sectional study. The study included patients under outpatient care or inpatient care of the rehabilitation medicine department. The inclusion criteria were as follows: (a) patients with SCI and (b) a functional ambulatory category score (FAC) of three points or less. Exclusion criteria were as follows: (a) history of bilateral total hip surgery, (b) any antiabsorptive or formative osteoporotic medication treatment, (c) parathyroid disease, and (d) untestable hip BMD due to heterotopic ossification or hip joint contracture. The exclusion criteria were set for patients for whom BMD could not be measured or who had a history of a disease or treatment that affected bone biomarkers.

The control group was recruited based on a retrospective chart review. We excluded patients with a medical history of cancer, liver pathology, kidney pathology, cerebral accident, or osteoporosis.

### 2.2. Assessments

The investigators evaluated BMI and bone biomarkers on the same day. Spine and hip BMD were assessed using dual-energy X-ray absorptiometry (Discovery Wi; Hologic Inc., Marlborough, MA, USA). Hip BMD had a lower value on both sides. The BMD of both the femoral neck and total femur was used to estimate the relationship with sclerostin. P1NP and CTX levels were analyzed in patient sera using automated electrochemiluminescence assays with the Cobas e601 apparatus (Roche Diagnostics, Mannheim, Germany). Serum sclerostin levels were measured using an enzyme-linked immunosorbent assay (ELISA) kit (Abcam; ab221836, Cambridge, UK). The tests were performed according to the manufacturer’s instructions, and a microplate reader (Spectramax 190; Molecular Devices, Sunnyvale, CA, USA) was used to read the ELISA plates.

Duration (in months) was defined as the difference between SCI and examination day of BMI and bone biomarkers. Patients were divided into two groups (acute and chronic) based on the injury duration (12 months) to investigate the effect of that duration on bone biomarker levels.

The FAC is a six-point walking scoring system. A score of 0 indicates a patient who is unable to move functionally. A score of 1 point indicates a patient who requires continuous manual contact to support body weight, to maintain balance, and for coordination; a score of 2 points indicates a patient who needs intermittent or continuous light touch; and a score of 3 points indicates a patient who can ambulate on a level surface without the physical help of another person but requires a person on standby for safety or verbal cueing. This study included patients with a score of 3 or less.

Lower extremity motor score (LEMS), as defined by the American Spinal Injury Association [17], is the sum of the key-muscle motor scores of the lower extremities. The key muscles of the lower extremities are the hip flexor, knee extensor, ankle dorsiflexor, long toe extensor, and ankle plantar flexor muscles. The motor score was the manual muscle-testing score for each key muscle. Even with a high LEMS, functional ambulation can be impossible due to sensory impairment. Therefore, we evaluated the relationship between lower extremity muscle strength through LEMS and bone biomarkers.

### 2.3. Statistical Analysis

The sample size was calculated by assuming an effect size of 0.3, a significance level of 0.05, and a power of 80% in the correlation analysis between sclerostin and duration. Data input and analyses were performed using SPSS (SPSS Inc., Chicago, IL, USA). The *t*-test and Mann–Whitney U test were used to evaluate differences between the acute and chronic groups based on the normality test. Moreover, the *t*-test and Mann–Whitney U test were also used to examine the differences between the sexes. Univariate regression analysis was used to estimate the factors that significantly affected sclerostin serum concentrations in patients with SCI. Statistical significance was set at *P* < 0.05.

## 3. Results

Sixty-two patients with SCI were recruited for this study (Table 1). Patients with prior osteoporosis medication were excluded. Moreover, patients for whom BMD examination could not be performed due to both side hip contractures were also excluded. Thirty-eight subjects were included in the control group.

The mean duration of injury was 93.58 ± 116.70 months. The mean durations of injury in the acute and chronic groups were 2.82 ± 2.38 and 155.28 ± 118.72 months, respectively. The bone biomarker levels are shown in Table 2 according to the sex of the patients, for both the SCI and control groups. Female patients with SCI showed a significantly higher concentration of P1NP than male patients. In addition, women in the control group showed higher concentrations of CTX than the men in the group.

The acute and chronic groups showed significant differences in age, duration, LEMS, bone biomarker levels (CTX, P1NP, and sclerostin), and BMD (Table 1 and Table 3).

The serum bone biomarker concentrations were significantly higher in the acute group than in the chronic group. All components of the bone biomarkers, including bone formation, bone absorption, and sclerostin, were significantly higher in the acute group than in the chronic group. Therefore, the acute group showed a high bone turnover. The hip BMD was lower in the chronic group than in the acute group.

**Figure 1:**
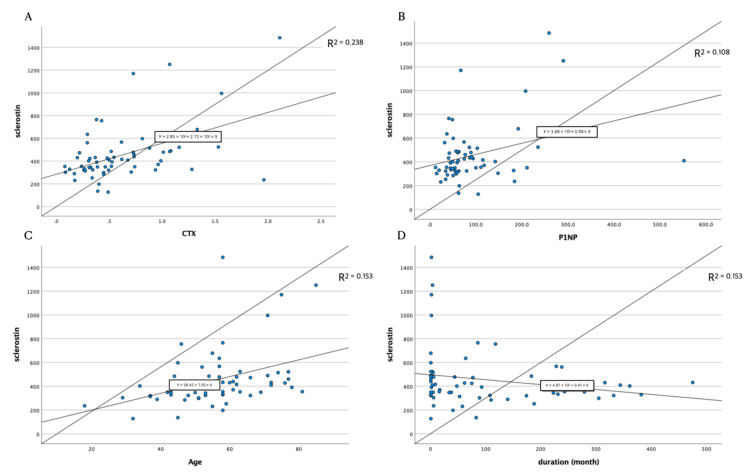
Scatterplot of sclerostin levels with bone biomarker levels, age, and duration. Bone biomarkers (**A**,**B**) and age (**C**) showed a positive relationship with sclerostin. However, the duration did not establish a significant relationship with sclerostin (**D**). In addition, there was a wide range variation of sclerostin serum concentration in the acute stage.

Age, CTX level, P1NP level, and BMD significantly affected the serum sclerostin concentration. Table 4 shows that CTX level, P1NP level, and age positively correlated with sclerostin levels (Figure 1).

**Figure 2:**
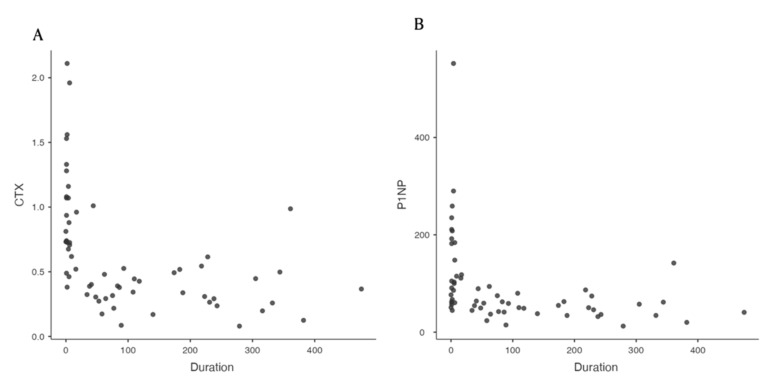
The scatterplot of bone biomarker levels against duration. Serum concentrations of carboxy-terminal collagen crosslinks (**A**) and procollagen type 1 intact N-terminal propeptide (**B**) were higher in the acute phase than in the chronic phase.

However, the duration of injury did not show a positive relationship with sclerostin (Figure 2).

In the early phase of SCI, sclerostin was present at higher serum concentrations than in the later phase. In the early phase, there was high bone turnover, followed by gradual stabilization.

## 4. Discussion

Patients with SCI who were unable to walk showed significantly higher serum bone biomarkers in the acute stage than in the chronic stage. Bone absorption and formation marker levels increased simultaneously, resulting in increased bone turnover. Moreover, sclerostin levels followed the trend of bone biomarker levels, with high levels in the acute stage and low levels in the chronic stage. Bone biomarker levels, age, and BMD independently and significantly affected sclerostin serum concentration.

Osteoporosis is a clinically significant medical issue in chronic SCI owing to the risk of osteoporotic fractures. Individuals with SCI who cannot walk are particularly vulnerable to osteoporosis in their lower extremities. Unloading is an essential factor for bone metabolism [18]. Unloading increases sclerostin, an inhibitor of bone formation, and can also reduce bone mineralization. Moreover, sclerostin reduces proliferation and increases apoptosis of osteoblasts [19]. Based on these notions, osteocytes act as mechanoreceptors, transforming mechanical strain into biochemical signaling [20]. There are few chances of strain or loading on the lower extremities in sedentary patients with SCI, which increases their risk of osteoporosis. Therefore, studies concerning sclerostin can increase our understanding of the pathophysiology and course of treatment for osteoporosis in patients with SCI.

Sedentary patients with SCI use a wheelchair to move, straining the spine instead of the lower extremities. This difference in loading and strain produces a dissociation between hip and lumbar spine demineralization [21]. Therefore, the BMD of the hips and knees shows a significant decrease compared to that of the spine. Moreover, the BMD of the distal femur is significantly correlated with the duration of SCI [22]. In our study, the acute and chronic groups showed significant differences in the hip BMD. The strain on the bone itself influences bone metabolism [23]. Moreover, strain and unloading influence sclerostin production [14]. Therefore, unloading and decreased strain have an overall effect on the bone demineralization of the lower extremities.

Following SCI, bone absorption (CTX) and bone formation (P1NP) simultaneously increase. Traditionally, osteoporosis has been thought to be caused by increased bone absorption. However, osteoclasts and osteoblasts communicate with each other, resulting in coupling activation, in which osteoclast activation stimulates osteoblast activation [24]. Hence, an increase in bone remodeling decreases bone mass and weakens bone strength [25]. Bone remodeling is the repair mechanism for stress damage in old bone tissue. Therefore, regular remodeling activity strengthens bone tissue to reduce fracture risk. However, higher bone remodeling marker levels decrease bone integrity and mineralization in newly synthesized bone [26]. In our study, bone turnover markers showed higher serum concentrations in the acute phase than in the chronic phase, indicating a higher bone turnover in the acute phase than in the chronic phase. Based on the bone biomarker levels in the control group, bone remodeling during the acute phase was remarkable. This highly acute bony remodeling decreases the resultant BMD in the chronic phase.

Osteocytes produce sclerostin, which impedes bone formation by inhibiting the Wnt signaling pathway in osteoblasts [27]. Long-term immobilization and unloading increase sclerostin concentrations [28]. Neurological deterioration following SCI makes patients vulnerable to immobilization and unloading. Therefore, sedentary SCI may be associated with high serum sclerostin levels that are related to osteoporosis. However, patients who use a wheelchair have significantly lower sclerostin levels than patients with chronic SCI who can walk on their own [29]. Patients with SCI with <5 years post-injury showed higher sclerostin concentrations than those with >5 years post-injury [30]. In our study, the acute phase (within 1 year post-injury) also showed a significant increase in sclerostin levels. The acute group showed variability in serum sclerostin concentration. Although unloading remarkably affects sclerostin levels, it does not cause a sustained elevation in sclerostin levels. Unloading in combination with age, BMD, and bone remodeling influences the serum concentration of sclerostin.

Osteocytes originate from osteoblasts following bone formation in the mineralized bone matrix [31]. Embedded osteocytes communicate with osteoblasts through the canaliculi. Decreased bone formation lowers the available pool of mature osteocytes and reduces the potential for sclerostin production. BMD showed a significant positive correlation with sclerostin levels; therefore, SCI patients with severe osteoporosis should have lower sclerostin levels [30,32]. The chronic group showed decreased BMD and thus lower sclerostin levels than the acute group. Moreover, the hip BMD was significantly correlated with sclerostin concentrations in the univariate regression analysis.

Age was positively correlated with the sclerostin levels. The level of sclerostin is affected not only by bone metabolism but also by medical conditions related to aging. The degree of osteoarthritis can affect the sclerostin levels [33]. Early stages of osteoarthritis increase sclerostin-positive osteocytes in the subchondral bone. However, in advanced osteoarthritis, the number of sclerostin-positive osteocytes decreases. Sclerostin levels are strongly correlated with plasma insulin and insulin resistance [34]. SCI carries the risk of insulin resistance due to reduced physical activity [35]. Therefore, glucose intolerance in older patients with SCI can increase sclerostin levels. Decreased glomerular filtration rate (GFR) also increases sclerostin levels [36]. Moreover, there is a positive relationship between serum phosphate and sclerostin levels, independent of GFR. Patients with chronic SCI are vulnerable to deterioration of the kidney function. These factors explain the positive correlation between aging and sclerostin levels.

In a rat SCI model, a sclerostin antibody prevented bone loss in the acute phase and preserved sublesional bone density in chronic conditions [37,38]. In this study, sclerostin concentration and bone remodeling were higher in the acute phase than in the chronic phase. Considering the high sclerostin levels in the acute phase, sclerostin antibody treatment may be promising for the prevention of bone loss in acute SCI. Moreover, sclerostin levels positively correlated with age. Clinical trials are needed to estimate the safety and efficacy of sclerostin antibody treatment in aging patients with SCI.

This study had some limitations. First, the patients were recruited from a single center. We did not conduct a follow-up study of the bone biomarker levels and only showed their cross-sectional results. Therefore, it was not sufficient to show the quantitative changes in the patients over time. Second, nutritional status and spasticity might significantly influence bone mineral density. However, we did not consider these factors in the analysis of bone metabolism.

## 5. Conclusions

The results of this study indicate sclerostin levels are high in the acute phase within 1 year of SCI and age, hip BMD, and bone remodeling are positively correlated with sclerostin levels in sedentary patients with SCI. Data regarding clinical factors affecting sclerostin concentration could help with the strategic development of preventative strategies or treatment options for osteoporosis in patients with SCI.

## Figures and Tables

**Table 1:** Demographic data.

		Total	Acute	Chronic	*P*
		Frequency			
Sex (male: female)		51:11	17:7	34:4	0.34
FAC (0:1:2:3)		44:12:3:3	14:5:3:2	30:4:0:1	
		Mean ± SD			
Age (years)		56.11 ± 13.21	57.41 ± 17.50	55.29 ± 9.83	0.47
Duration (months)		100.68 ± 122.36	2.82 ± 2.38	155.28 ± 118.72	0.00 *
BMI (kg/m^2^)		22.46 ± 3.80	23.77 ± 4.63	21.63 ± 2.95	0.12
LEMS		11.40 ± 13.69	20.73 ± 14.58	5.54 ± 9.27	0.00 *
Injured spinal level	Cervical	33	17	16	
	Thoracic	23	5	18	
	Lumbar	6	2	4	

* *P* < 0.05. *P*-values were calculated between the acute and chronic groups and analyzed using *t*-test and Mann–Whitney analysis. LEMS, lower extremity motor score; BMI, body mass index; FAC, functional ambulatory score; SD, standard deviation.

**Table 2:** Bone biomarker levels according to the sex.

Spinal cord injury		**Total (*n* = 62)**	**Male (*n* = 51)**	**Female (*n* = 12)**	***P***
	Age (years)	56.11 ± 13.21	56.90 ± 12.33	53.58 ± 17.66	0.48
	CTX (ng/mL)	0.59 ± 0.39	0.62 ± 0.45	0.65 ± 0.36	0.42
	P1NP (ng/mL)	86.02 ± 80.98	79.74 ± 60.58	152.31 ± 147.05	0.03 *
Control group		**Total (*n* = 96)**	**Male (*n* = 34)**	**Female (*n* = 62)**	
	Age (years)	59.89 ± 6.01	60.82 ± 6.74	59.37 ± 5.55	0.25
	CTX (ng/mL)	0.44 ± 0.26	0.36 ± 0.22	0.49 ± 0.26	0.02 *
	P1NP (ng/mL)	50.39 ± 24.43	51.38 ± 23.72	50.07 ± 24.99	0.80

* *P* < 0.05. Statistical analyses were performed to determine the differences between men and women. CTX, carboxy-terminal collagen crosslinks; P1NP, procollagen type 1 intact N-terminal propeptide.

**Table 3:** Bone biomarker levels and bone mineral density.

		Total	Acute	Chronic	*P*
Bone biomarkers	CTX (ng/mL)	0.59 ± 0.39	0.99 ± 0.45	0.40 ± 0.21	0.00 *
	P1NP (ng/mL)	86.02 ± 80.98	147.54 ± 112.70	56.84 ± 28.25	0.00 *
	Sclerostin (pg/mL)	426.35 ± 182.67	555.28 ± 335.20	390.72 ± 133.94	0.02 *
BMD (g/cm^2^)	Right femur neck	0.66 ± 0.15	0.76 ± 0.10	0.59 ± 0.13	0.00 *
	Right femur total	0.77 ± 0.16	0.91 ± 0.11	0.69 ± 0.13	0.00 *
	Left femur neck	0.66 ± 0.15	0.76 ± 0.14	0.60 ± 0.12	0.00 *
	Left femur total	0.74 ± 0.16	0.86 ± 0.13	0.67 ± 0.13	0.00 *

* *P* < 0.05. CTX, carboxy-terminal collagen crosslinks; P1NP, procollagen type 1 intact N-terminal propeptide; BMD, bone mineral density.

**Table 4:** Univariate regression analysis of clinical factors according to sclerostin serum concentration.

		B	SE	β	*P*
Age		7.05	2.14	0.39	0.00 *
CTX		2713.98	62.89	0.48	0.00 *
P1NP		0.94	0.35	0.32	0.01 *
LEMS		3.34	2.30	0.18	0.15
BMI		10.21	8.47	0.15	0.23
Duration		−0.41	0.25	−0.20	0.10
BMD	Right femur neck	580.12	193.22	0.36	0.00 *
	Right femur total	602.64	171.41	0.41	0.00 *
	Left femur neck	326.52	149.12	0.27	0.03 *
	Left femur total	425.28	129.91	0.39	0.00 *

* *P* < 0.05. LEMS, lower extremity motor score; CTX, carboxy-terminal collagen crosslinks; P1NP, procollagen type 1 intact N-terminal propeptide; BMD, bone mineral density.

## Data Availability

The dataset used and/or analyzed during the current study area is available from the corresponding author upon reasonable request.
